# Wild ungulate species differ in their contribution to the transmission of *Ixodes ricinus*-borne pathogens

**DOI:** 10.1186/s13071-021-04860-w

**Published:** 2021-07-10

**Authors:** Nannet D. Fabri, Hein Sprong, Tim R. Hofmeester, Hans Heesterbeek, Björn F. Donnars, Fredrik Widemo, Frauke Ecke, Joris P. G. M. Cromsigt

**Affiliations:** 1 Department of Wildlife, Fish, and Environmental Studies, Swedish University of Agricultural Sciences, 901 83 Umeå, Sweden; 2 Department of Population Health Sciences, Faculty of Veterinary Medicine, Utrecht University, Yalelaan 7, 3584 CL Utrecht, The Netherlands; 3 Centre for Infectious Disease Control, National Institute for Public Health and the Environment, Antonie Van Leeuwenhoeklaan 9, 3721 MA Bilthoven, The Netherlands; 4 Centre for African Conservation Ecology, Department of Zoology, Nelson Mandela University, PO Box 77000, Port Elizabeth, 6031 South Africa; 5 Copernicus Institute of Sustainable Development, Faculty of Geosciences, Utrecht University, Princetonlaan 8a, 3584 CB Utrecht, The Netherlands

**Keywords:** *Anaplasma phagocytophilum*, *Borrelia burgdorferi* (*s.l.*), *Ixodes ricinus*, Ungulate management, Zoonotic disease risk

## Abstract

**Background:**

Several ungulate species are feeding and propagation hosts for the tick *Ixodes ricinus* as well as hosts to a wide range of zoonotic pathogens. Here, we focus on *Anaplasma phagocytophilum* and *Borrelia burgdorferi* (*s.l.*), two important pathogens for which ungulates are amplifying and dilution hosts, respectively. Ungulate management is one of the main tools to mitigate human health risks associated with these tick-borne pathogens. Across Europe, different species of ungulates are expanding their ranges and increasing in numbers. It is currently unclear if and how the relative contribution to the life-cycle of *I. ricinus* and the transmission cycles of tick-borne pathogens differ among these species. In this study, we aimed to identify these relative contributions for five European ungulate species.

**Methods:**

We quantified the tick load and collected ticks and spleen samples from hunted fallow deer (*Dama dama*, *n* = 131), moose (*Alces alces*, *n* = 15), red deer (*Cervus elaphus*, *n* = 61), roe deer (*Capreolus capreolus*, *n* = 30) and wild boar (*Sus scrofa*, *n* = 87) in south-central Sweden. We investigated the presence of tick-borne pathogens in ticks and spleen samples using real-time PCR. We determined if ungulate species differed in tick load (prevalence and intensity) and in infection prevalence in their tissue as well as in the ticks feeding on them.

**Results:**

Wild boar hosted fewer adult female ticks than any of the deer species, indicating that deer are more important as propagation hosts. Among the deer species, moose had the lowest number of female ticks, while there was no difference among the other deer species. Given the low number of infected nymphs, the relative contribution of all ungulate species to the transmission of *B. burgdorferi* (*s.l.*) was low. Fallow deer, red deer and roe deer contributed more to the transmission of *A. phagocytophilum* than wild boar.

**Conclusions:**

The ungulate species clearly differed in their role as a propagation host and in the transmission of *B. burgdorferi* and *A. phagocytophilum*. This study provides crucial information for ungulate management as a tool to mitigate zoonotic disease risk and argues for adapting management approaches to the local ungulate species composition and the pathogen(s) of concern.

**Graphic abstract:**

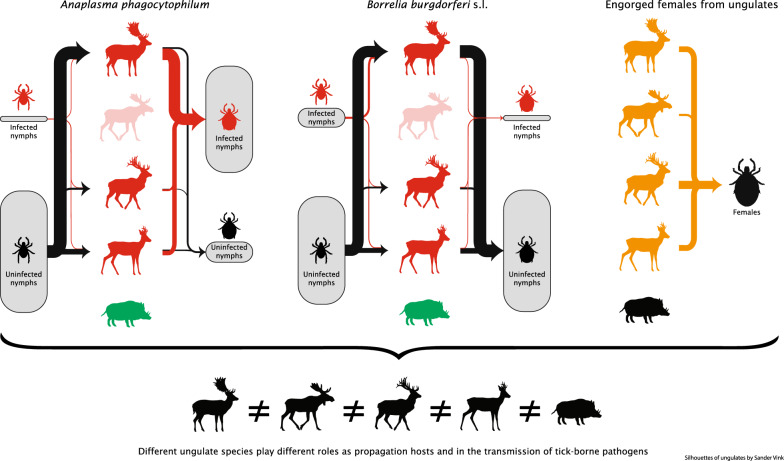

**Supplementary Information:**

The online version contains supplementary material available at 10.1186/s13071-021-04860-w.

## Background

Wild ungulates are common across Europe, and several ungulate species have increased their densities and expanded their ranges during the last decades [1–3]. These changes can be attributed to improved protection, the absence of large carnivores in certain areas, food subsidies due to new agricultural and forestry practices and less severe winters [2, 4, 5]. As a result, many areas in Europe currently host a higher diversity of ungulate species than during the recent past [6]. This increase of wild ungulates has allowed their ectoparasites, such as the tick species *Ixodes ricinus*, to increase in densities and expand their ranges [7], leading to an increase in the prevalence of tick-borne zoonotic pathogens, such as *Anaplasma phagocytophilum* and *Borrelia burgdorferi* (*s.l.*). Ungulates play a central role in the life-cycle of *I. ricinus* as feeding hosts, but most importantly as propagation hosts [8]. However, it is poorly understood if and how ungulate species differ in terms of their relative contribution to the tick life-cycle and to the transmission of tick-borne pathogens. Although several studies have looked at the role of ungulates in tick-borne pathogen transmission, only a few of these studied multiple ungulate species simultaneously (e.g. [9–13]). Furthermore, data on certain common ungulate species, particularly wild boar and fallow deer, are currently still scarce [8].

There are several ways in which an ungulate can contribute to the abundance of infected ticks. Two main pathways are: (i) a tick becomes infected while feeding on an ungulate and (ii) an infected tick feeds on an ungulate (regardless of infection status) and detaches fully engorged and still infected. Ungulates also influence the local abundance of infected ticks through their movement, since they might spread the infected ticks to other areas. Aspects that are relevant for pathogen transmission from an infected ungulate to an uninfected tick include the presence of the pathogen in the ungulate, the reservoir competence of the ungulate and the transmission rate of the pathogen from the ungulate to the tick. Ungulates are considered competent hosts for *A. phagocytophilum*, and all European ungulate species can become infected with the pathogen, as has been shown in several studies (reviewed in [14]). However, it is unclear if these species differ in terms of their role in the transmission of *A. phagocytophilum*. Ungulates are not considered to be competent hosts for *B. burgdorferi* (*s.l.*), and it is therefore unlikely that they can transmit this pathogen to ticks [8]. Indeed, it has been proposed that ungulates can have a negative (borreliacidal) effect on the presence of *B. burgdorferi* (*s.l.*) in ticks [9], although the potential impact of this borreliacidal effect remains unclear.

Common and widespread European ungulate species include roe deer (*Capreolus capreolus*), wild boar (*Sus scrofa*), red deer (*Cervus elaphus*) and fallow deer (*Dama dama*) [6]; in northern Europe, the moose (*Alces alces*) is also widespread and abundant. These species have different morphological and behavioral traits (Table 1), which may influence the likelihood of an ungulate encountering a tick, a tick attaching to an ungulate or an engorged (and infected) tick detaching. For example, variation in leg length among ungulate species may affect the likelihood of attachment by ticks because leg length influences the distance that an adult tick has to travel to preferred feeding sites, such as the axilla and groin [9, 15], and variation in hair structure and skin thickness will likely influence the potential for ticks to penetrate the skin and find a blood meal. Ungulate feeding behavior may also be important in this context since it will be easier for nymphs and larvae to attach to the ears of species that predominately feed in the field layer, such as fallow deer, than to the ears of species that browse higher up, such as moose [9, 15]. In terms of social behavior, grooming behavior and wallowing influence the ability of a tick to fully complete its blood meal, and herd size may influence the likelihood of encountering a tick.

**Table 1:** Several traits of five ungulate species

Trait	Fallow deer	Moose	Red deer	Roe deer	Wild boar	References
Body mass (kg)	57	462	240	23	84	[16]
Home range (km^2^)	0.7	71.8	54.8	0.5	1.2	[16]
Diet	Grass, fruits and seeds	Trees, shrubs	Trees, shrubs, forbs, grass	Trees, shrubs, crops	Fruits and seeds, grass, crops	[17]
Social structure	Gregarious (big groups)	Solitary	Gregarious (small groups)	Small family groups	Gregarious (big groups)	

In this study, performed in south-central Sweden, we collected ticks and spleen samples from five common and sympatric European ungulate species, with the aim to determine tick burdens and the prevalence of *A. phagocytophilum* and *B. burgdorferi* (*s.l.*). Based on variation in the aforementioned traits (Table 1), we hypothesized that the ungulate species would differ in their relative contribution as propagation host as well as their role in the transmission of *A. phagocytophilum* and *B. burgdorferi* (*s.l.*). We also present the infection prevalence of *A. phagocytophilum*, *B. burgdorferi* (*s.l.*), *Borrelia miyamotoi* and *Babesia* spp. for the five ungulate species, the infection prevalence of these pathogens in engorged ticks collected from the ungulates and the infection prevalence in questing nymphs and adults.

## Methods

### Sample collection

We opportunistically collected ticks and spleen samples from ungulates shot by hunters on hunting estates in three counties in south-central Sweden: Södermanland, the southernmost part of Stockholm county and the western part of Östergötland (Additional file 1: Figure S1). We selected these areas as they host the most diverse and abundant ungulate populations in Sweden and fall within Sweden’s climatic zone where ticks can be abundant [7, 18, 19]. The local habitat is characterized by forests dominated by Scots pine (*Pinus sylvestris*), Norway spruce (*Picea abies*), birch (*Betula* spp.) and European oak (*Quercus robur*), interspersed with agricultural lands with diverse crops [18]. We sampled a total of 324 ungulates: 131 fallow deer, 15 moose, 61 red deer, 30 roe deer and 87 wild boars during October 2018 and October and November 2019. However, not all individuals were sampled for both spleen and ticks (see Additional file 1: Table S1 for a detailed overview).

**Fig. 1:**
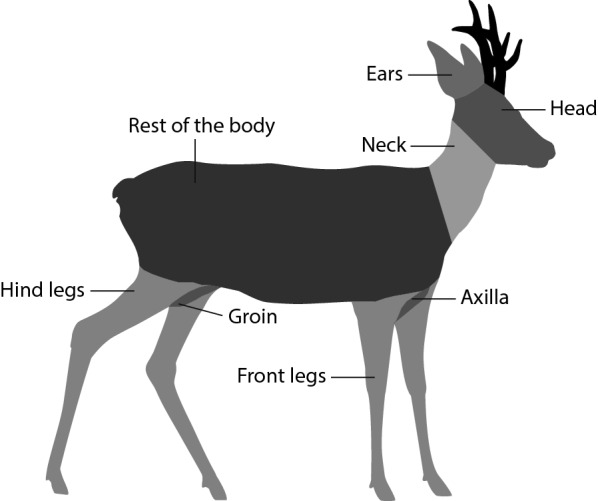
Illustration of the tick collection sites on roe deer. For the other four ungulate species, we used the same tick collection sites (see Additional file 1: Table S2). Figure is adapted from Kiffner et al. [15]. Silhouette by Sander Vink

Hunters gutted each ungulate almost directly after they shot it and, if possible, gave us a part of the spleen. We sampled spleens from the ungulates (in contrast to, for example, sampling blood) since the spleen was easy to collect by the hunters involved in our study and since spleens allow for the detection of multiple tick-borne pathogens simultaneously [11, 20, 21]. Of the 305 animals from which we collected ticks, 182 were checked immediately after gutting; the other 123 animals were stored in cooling chambers (2–6 °C) after gutting and checked 1–6 days after they were shot. Ticks that fell off during this period were not collected. To correct for this, we included the number of days, from the moment the animals were shot until the moment the animals were checked for ticks, in the statistical analysis. We counted the number of ticks separately for eight different body parts (Fig. 1; adjusted from Kiffner et al. [15]). We used forceps to remove all counted ticks and recorded tick life stage and sex, from which part of the body it was collected and whether it was attached, walking or attached to a female (the latter only for males). Furthermore, we recorded the sex and age of the ungulate, and the estate where the animal was shot. We kept all ticks from the same ungulate individual in two sampling tubes with 70% ethanol (one for feeding and one for non-feeding ticks) and stored these at − 20 °C until analysis in the laboratory. Ticks were morphologically identified to species level using morphological keys as described in [22, 23], and all were determined to be *I. ricinus*. This was confirmed microscopically for approximately 30% (*n* = 994) of the ticks.

In addition to the ticks collected directly from the animals, we also collected questing nymphs (*n* = 881) and adults (*n* = 84) by dragging a 1-m^2^ white cotton cloth over the vegetation in the same areas as where the ungulates were shot. The questing ticks also included ticks found on researchers during dragging (*n* = 54). We collected the questing ticks in September 2018 and May–August 2019, similar to the period our sampled ungulates were shot. We counted these questing ticks separately for each life stage and sex (nymphs, males and females) and morphologically identified them to species following [22, 23]. Again, we confirmed our initial morphological determination microscopically for 30% of the individuals (*n* = 291). Of the questing ticks, one male was identified as *Haemaphysalis punctata*, while all others were identified as *I. ricinus.* Sex was not recorded for the adults collected in 2018. We stored the questing ticks individually in 8-strip Eppendorf tubes® (Eppendorf AG, Hamburg, Germany) at − 20 °C.

### DNA extraction and pathogen detection

DNA was extracted from unengorged and questing ticks with ammonium hydroxide as described in [24], and DNA was extracted from engorged ticks and spleen samples using the Qiagen DNeasy Blood & Tissue Kit according to the manufacturer’s protocol (Qiagen, Hilden, Germany). We stored the lysates at 4 °C until further analysis. For pathogen detection we used multiplex real-time PCRs on various targeting genes for *A. phagocytophilum* [25], *B. burgdorferi* (*s.l.*) [26], *B. miyamotoi* [27], *Babesia microti* [28] and *Babesia-*clade X [29]. We followed a qPCR protocol as described in [28]. We amplified all *A. phagocytophilum*-positive spleen samples and 58 of the ticks collected from ungulates that were positive for *A. phagocytophilum* by conventional PCR followed by sequencing to identify an ecotype [25]. Of the ticks collected from ungulates that were positive for *B. burgdorferi* (*s.l.*), we amplified 198 by conventional PCR followed by sequencing to identify the genotype [26]. We did the same for all *Babesia*-positive spleen samples and 64 of the ticks collected from ungulates that were positive for *Babesia* spp. [30]. We could not amplify and sequence all positive ticks due to practical constraints, but previous work has indicated that these sample sizes are representative of the whole population [8]. Furthermore, we did not amplify and type material from any positive questing ticks. 

### Using body parts as proxy for the whole animal to increase sample sizes

Some of the carcasses were not complete at the moment of tick collection (Additional file 1: Table S1) due to actions by the hunters. We assessed how the number of ticks on certain body parts correlated with the number of ticks found on the whole animal. For this, we included 261 animals for which we checked the complete body for ticks. Of the total number of feeding ticks found on these animals (52 larvae, 1233 nymphs and 966 females), all larvae and > 90% of the nymphs were on the ears, while we found > 90% of the females on the axilla and groin combined (Additional file 1: Table S2). As a result, there was a strong linear correlation between the number of nymphs found on the whole body* versus* on the ears (*R*^2^_adj_ = 0.999, *P* < 0.001), and between the number of females found on the whole body* versus* on the axilla and groin combined (*R*^2^_adj_ = 0.987, *P* < 0.001). Consequently, in our further analyses, we used the larval and nymphal infestation on the ears as proxies for the total larval and nymphal infestation, respectively, and the female infestation on the groin and axilla combined as a proxy for the total female infestation. This allowed us to increase the sample sizes for our statistical analyses. For the non-feeding male infestation, we only used the ungulate individuals for which we had a full body count, since the males were not attached to their host and therefore not bound to a specific body part.

### Contribution of ungulate species as a propagation host

**Fig. 2:**
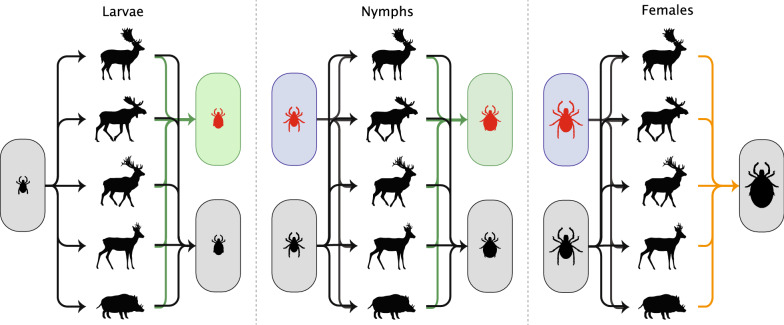
Theoretical framework on how *Ixodes ricinus* can feed on ungulates and become infected. The arrows from questing ticks to ungulates show the attachment routes, and the arrows from ungulates to engorged ticks show detachment routes. Red ticks are infected ticks, either for *Anaplasma phagocytophilum* or for *Borrelia burgdorferi* (*s.l.*). The engorged females are not divided into infected and uninfected, since we assume that there is no vertical transmission and thus the infection status of an engorged female is irrelevant. The green arrows are the detachment routes of infected larvae and nymphs and show the role of the ungulate species in the transmission of either *A. phagocytophilum* or *B. burgdorferi* (*s.l.*). The orange arrows are the detachment routes of engorged females and show the role of the ungulate species as propagation host. Green boxes show the infection prevalence of engorged ticks, while blue boxes show the infection prevalence in questing ticks. Silhouettes of ungulates by Sander Vink

We determined the contribution of each ungulate species as a propagation host by determining the tick burden and the infestation prevalence for female ticks and the infestation prevalence for non-feeding male ticks (depicted by the orange lines in Fig. 2). We calculated the tick burden of female ticks using:

1 $${{T}}_{{{{F}}_{i} }} = {{P}}_{{{{F}}_{i} }} \cdot {{I}}_{{{{F}}_{i} }}$$

where ${{T}}_{{{{F}}_{i} }}$ is the female tick burden on host species $i$, ${{P}}_{{{{F}}_{i} }}$ is the infestation prevalence of females in host species $i$and ${{I}}_{{{{F}}_{i} }}$ is the infestation intensity of females in host species $i$. Following Kahl et al. [31] we defined the mean infestation prevalence as the proportion of hosts with feeding ticks on the body parts described above, and the mean infestation intensity as the number of ticks feeding on those body parts, for those hosts that had feeding ticks. For both parameters, we estimated a 95% bootstrapped, bias-corrected, confidence interval (BCa-CI). To calculate the female tick burden, we used the predicted values from the models for the infestation prevalence and intensity of females, which were obtained as described below. To assess differences among the ungulate species in the female tick burden, we compared the 84% bootstrapped, bias-corrected confidence intervals with each other to obtain a significance with an alpha value of 0.05, as suggested by Payton et al. [32].

To obtain predicted values and to test for possible differences among ungulates in the infestation prevalence of females and non-feeding males and the infestation intensity of females, we used hierarchical GLMMs that included, as fixed effects, ungulate species, ungulate sex (female or male), ungulate age group (adult or young) and the number of days between the day the animal was shot and when it was checked for ticks. For the models of infestation prevalence we also included the month of collection (October 2018, October or November 2019) as a fixed effect and a random effect for each hunting estate where the animal was shot (see Additional file 2 for these variables). For the models of infestation intensity, we excluded ungulate species with less than ten individuals infested, since the sample size would be too small. We included a combined random effect for each combination of month of collection and hunting estate due to unbalanced numbers of infested ungulates on the estates over the seasons. We split the hierarchical GLMM by first modeling the infestation prevalence using a GLMM with a binomial distribution. Then, we modeled the infestation intensity, using a GLMM with a zero-truncated negative binomial distribution on a subset of animals on which we found the female tick stage [33]. We fitted GLMMs using the glmmTMB package [34]. We performed model selection, starting with the full models with all above-described parameters as additive effects (i.e. no interactions) using the dredge function in the MuMIn-package. We selected the best fitting models based on the principle of Occam’s razor; i.e. from all models with differences in Akaike's information criterion (ΔAIC) < 4, we selected the models with the fewest variables [35].

### Contribution of ungulate species to the transmission of *Anaplasma phagocytophilum* and *Borrelia burgdorferi* (*s.l.*)

We determined the contribution of a host to the transmission of *A. phagocytophilum* and *B. burgdorferi* (*s.l.*) by quantifying the infection intensity of engorged larvae and nymphs on each ungulate species (depicted by the green lines in Fig. 2). We defined the infection intensity as the mean number of infected ticks found on an individual of each species during the tick-questing period, which is the time when the temperature is above 7 °C, roughly from May until October in our study area [36]. We focused on larvae and nymphs because they molt into infected ticks of the next stage and can ultimately infect another animal or human. We excluded engorged females because they do not produce any infected offspring for either *A. phagocytophilum* or *B. burgdorferi* (*s.l.*) [37–39]. The infection intensity was calculated as:

2 $$n_{{{{AL}}_{i} }} = {{P}}_{{{{L}}_{i} }} \cdot {{I}}_{{{{L}}_{i} }} \cdot {{S}}_{{{{LA}}_{i} }}$$

where $n_{{{{AL}}_{i} }}$ is the *A. phagocytophilum* infection intensity of engorged larvae from host species $i$, ${{P}}_{{{{L}}_{i} }}$ is the infestation prevalence of larvae from host species $i$, ${{I}}_{{{{L}}_{i} }}$ is the infestation intensity of larvae from host species $i$ and ${{S}}_{{{{LA}}_{i} }}$ is the *A. phagocytophilum* infection prevalence in larvae from host species $i$. The *B. burgdorferi* (*s.l.*) infection intensity of engorged larvae ($n_{{{{BL}}_{i} }}$), the *A. phagocytophilum* infection intensity of engorged nymphs ($n_{{{{AN}}_{i} }}$) and the *B. burgdorferi* (*s.l.*) infection intensity of engorged nymphs ($n_{{{{BN}}_{i} }}$) from host species $i$ can be calculated by substituting $A$ by $B$ and/or $L$ by $N$. We defined the infection prevalence as the proportion of infected ticks among all ticks collected from ungulates, for each tick-borne pathogen, ungulate host species and tick stage. We again estimated the 95% BCa-CIs for all parameters. To calculate the *A. phagocytophilum* and *B. burgdorferi* (*s.l.*) infection intensity of engorged nymphs, we used the predicted values from the models for infestation prevalence and intensity of nymphs and for the *A. phagocytophilum* and *B. burgdorferi* (*s.l.*) infection prevalence, which were obtained as described below. To assess differences among the ungulate species in the infection intensity of engorged larvae and nymphs, we compared the 84% bootstrapped, bias-corrected, confidence intervals with each other to obtain a significance with an alpha value of 0.05 [32].

We performed a Šidák-adjusted Dunn-test to establish if there were any differences in the prevalence of larval infestation, the intensity of larval infestation and the infection prevalence in engorged larvae among the ungulate species. We used this approach because of the small larval sample sizes (Additional file 1: Table S2). To test for possible differences in the infestation prevalence and intensity of nymphs among the ungulate species, we used hierarchical GLMMs, with the same model structure as described for females and non-feeding males. To test for an effect of ungulate species on the infection prevalence in engorged nymphs, we also used a GLMM with a binomial distribution. We included the same fixed effects as in the GLMM of the infestation intensity, however we excluded the number of days between the day the animal was shot and when it was checked for ticks, since this does not affect the infection status of a tick. We excluded ungulate species with less than ten nymphs tested, since the sample size would be too small. We included a random effect for each host nested within each combination of year of collection (2018 or 2019) and hunting estate.

### Pathogen transmission from ungulate host to ticks

To estimate the extent to which ungulate species can infect ticks that feed on them, we compared the infection prevalence of feeding nymphs, feeding females and non-feeding males with the infection prevalence of questing nymphs and questing adults, respectively, with the Šidák-adjusted Dunn-test, for *A. phagocytophilum*, *B. burgdorferi* (*s.l.*), *B. miyamotoi* and *Babesia* spp. We established that there was a difference between the ticks on animals and the questing ticks if the* P*-value was lower than half the alpha value of 0.05.

We performed all analyses in R version 3.6.0 [40] and used an alpha value of 0.05.

## Results

**Fig. 3:**
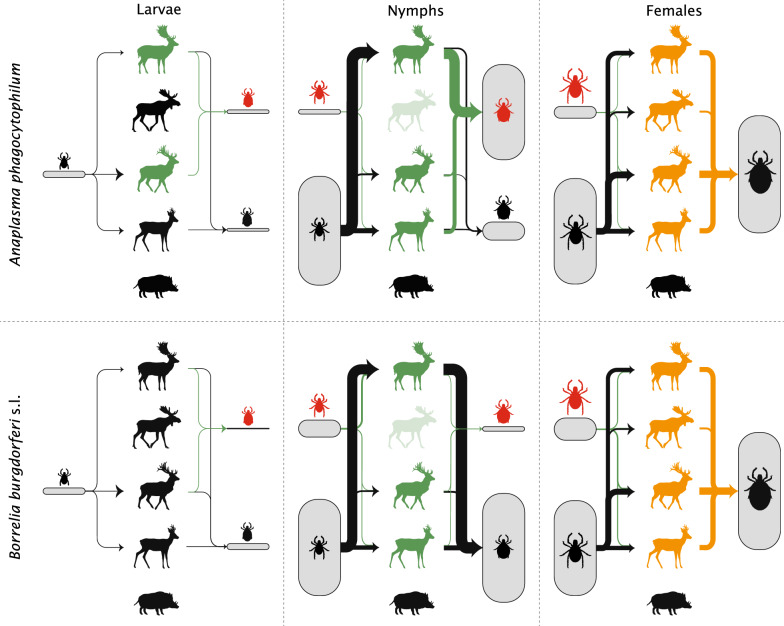
Illustration of the transmission of *Anaplasma phagocytophilum* and *Borrelia burgdorferi* (*s.l.*) by ungulate species. The arrows from questing ticks to ungulates show the attachment routes and the arrows from ungulates to engorged ticks show detachment routes. The thickness of the arrows represents the proportion of ticks attaching or detaching, and the size of the boxes represents the proportion of that tick stage, based on data from Table 2. Red ticks represent infected ticks. The engorged females are not divided into infected and uninfected, since we assume that there is no vertical transmission and thus the infection status of an engorged female is irrelevant. The green arrows and green ungulates show the role of the ungulate species in the transmission of either *A. phagocytophilum* or *B. burgdorferi* (*s.l.*), and the orange arrows and orange ungulates show the role of the ungulate species as propagation host. Light-green coloration of ungulate means that the role of this ungulate in the transmission of either *A. phagocytophilum* or *B. burgdorferi* (*s.l.*) is unknown. Silhouettes of ungulates by Sander Vink

The contribution of ungulates as propagation hosts to and the transmission of *Anaplasma phagocytophilum* and *Borrelia burgdorferi* (*s.l.*) varied among species (Fig. 3). We describe the results for the different pathways in detail in the following sections.

### Contribution of ungulate species as propagation hosts

Of the 261 ungulates of which the whole carcass was checked for ticks, 119 animals were infested with 515 non-feeding males in total (Additional file 1: Table S3). Based on the selected model (Additional file 1: Table S4), moose had the highest infestation prevalence of non-feeding males, and red deer the second highest. Wild boar had the lowest infestation prevalence of non-feeding males, and fallow deer the second lowest, while roe deer did not differ from either red deer or fallow deer (Table 2). We included 300 ungulates that were checked for female ticks on at least both groins and both axillae, where 192 animals were infested with 1179 females in total on the groin and axilla (Additional file 1: Table S3). Based on the selected models (Additional file 1: Table S5), all four deer species had a higher infestation prevalence of females than wild boar, and there was no difference among the deer species in terms of the infestation intensity of females (Table 2). For the model for infestation intensity of females, we excluded wild boar since only five of 82 individuals were infested with females on the groin and axilla (Additional file 1: Table S3). There was no difference in the female tick burden among the deer species (Table 2; Additional file 1: Figure S2), while a female tick burden for wild boar could not be calculated.

**Table 2:** Summary of the examined parameters for the five studied ungulate species and tick stage

Parameters	Feeding larvae		Feeding nymphs		Feeding females		Non–feeding males	
Infestation prevalence (95% CI)^a^								
Fallow deer	0.18 (0.11–0.26)	a	0.97 (0.91–0.99)	a	0.95 (0.81–0.99)	a	0.60 (0.40–0.77)	a
Moose	0.00^b^	a,b	0.39 (0.13–0.73)	b,c	0.98 (0.81–1.00)	a	1.00^b^	b
Red deer	0.04 (0.00–0.10)	b	0.66 (0.40–0.85)	b	0.97 (0.88–0.99)	a	0.98 (0.90–0.99)	c
Roe deer	0.10 (0.00–0.21)	a,b	0.94 (0.81–0.99)	a	0.96 (0.78–0.99)	a	0.84 (0.59–0.95)	a,c
Wild boar	0.00^b^	b	0.17 (0.07–0.36)	c	0.03 (0.01–0.17)	b	0.04 (0.01–0.16)	d
Infestation intensity (95% CI)^a^								
Fallow deer	2.47 (1.79–3.84)	a	8.89 (5.80–13.62)	a	3.88 (2.50–6.03)	a		
Moose	0.00		–^d^		1.43 (0.50–4.10)	a		
Red deer	2.50 (2.00–3.00)	a	2.07 (0.54–7.89)	a	5.49 (3.44–8.76)	a		
Roe deer	2.33 (1.00–3.33)	a	3.96 (1.63–9.63)	a	3.93 (2.30–6.71)	a		
Wild boar	0.00		–^d^		–^d^			
Tick burden (95% CI)								
Fallow deer	0.44 (0.17–1.07)	a	8.62 (4.91–14.33)	a	3.67 (1.83–6.37)	a		
Moose	0.00 b		–		1.40 (0.32–4.77)	a		
Red deer	0.10 (0.00–0.30)	b	1.37 (0.17–8.32)	a	5.33 (2.73–9.37)	a		
Roe deer	0.23 (0.00–0.96)	a,b	3.72 (1.12–10.83)	a	3.77 (1.53–7.25)	a		
Wild boar	0.00	b	–		–			
Infection prevalence *Anaplasma phagocytophilum *(95% CI)^a^								
Fallow deer	0.79 (0.62–0.88)	a	0.87 (0.77–0.93)	a				
Moose	0.00^c^	a,b	–^e^					
Red deer	1.00^c^	a	0.87 (0.64–0.96)	a				
Roe deer	0.00^c^	b	0.76 (0.54–0.89)	a				
Wild boar	–		–^e^					
Infection intensity *Anaplasma phagocytophilum* (95% CI)								
Fallow deer	0.35 (0.09–0.98)	a	7.50 (3.48–13.78)	a				
Moose	0.00	b	–					
Red deer	0.10 (0.00–0.36)	a,b	1.19 (0.08–8.95)	a				
Roe deer	0.00	b	2.83 (0.50–10.56)	a				
Wild boar	0.00	b	–					
Infection prevalence *Borrelia burgdorferi * (*s.l.*) (95% CI)^a^								
Fallow deer	0.04 (0.00–0.10)	a	0.04 (0.02–0.06)	a				
Moose	0.00^c^	a	–^e^					
Red deer	0.20 (0.00–0.40)	a	0.04 (0.01–0.12)	a				
Roe deer	0.00^c^	a	0.04 (0.01–0.09)	a				
Wild boar	–		–^e^					
Infection intensity *Borrelia burgdorferi* (*s.l.*) (95% CI)								
Fallow deer	0.02 (0.00–0.15)	a	0.31 (0.09–0.89)	a				
Moose	0.00	a	–					
Red deer	0.02 (0.00–0.22)	a	0.05 (0.00–1.29)	a				
Roe deer	0.00	a	0.15 (0.01–1.28)	a				
Wild boar	0.00	a	–					

All values for infestation prevalence, infestation intensity and infection prevalence are predicted values from the models in our study, except for the larvae. 95% confidence intervals (CI) are given in parentheses. The lowercase letters indicate the significant differences among the ungulate species

^a^The 95% CI for the infestation prevalence, infestation intensity and infection prevalence are 95% bootstrapped, bias-corrected confidence intervals

^b^The CI for infestation prevalence cannot be calculated if none or all of the animals were infested

^c^The CI for infection prevalence cannot be calculated if none or all of the ticks were infected

^d^The predicted values of the infestation intensity cannot not be obtained due to low number of animals

^e^The predicted values of the infection prevalence cannot be obtained due to low number of tested ticks

### Infection prevalence of tick-borne pathogens in ungulates

None of the investigated spleen samples from 64 fallow deer, eight moose, 28 red deer, seven roe deer and 34 wild boars were positive for *B. burgdorferi* (*s.l.*) or *B. miyamotoi*. *Anaplasma phagocytophilum* was found in all ungulate species, although the prevalence was lower in wild boar (Table 3). We determined the ecotype of *A. phagocytophilum* through sequencing of 43 fallow deer, two moose, 20 red deer, seven roe deer and seven wild boars: ecotype 2 was found in all roe deer, while all the other ungulate species harbored ecotype 1. None of the ungulates tested positive for *B. microti*. *Babesia* (*s.s.*) was found in the deer species, but not in wild boar (Table 3). The *Babesia* spp. was determined through sequencing of five fallow deer, three moose, 16 red deer and seven roe deer samples: *B. capreoli* was found in fallow deer and roe deer, *B. divergens* in fallow deer, red deer and roe deer, *B. odocoilei-EU* in fallow deer, moose and red deer and *B. venatorum* in red deer and roe deer (Table 3).

**Table 3:** Infection prevalence of tick-borne pathogens in the five studied ungulate species

Ungulate species	*Anaplasma phagocytophilum*		*Borrelia burgdorferi* (*s.l.*)		*Borrelia miyamotoi*		*Babesia* spp.	
	*n*_P_	IP (95% CI)	*n*_P_	IP (95% CI)	*n*_P_	IP (95% CI)	*n*_P_	IP (95% CI)
Fallow deer (*n* = 65)	63^a^	0.98 (0.92–1.00)	0	0.00	0	0.00	9^c^	0.14 (0.06–0.23)
Moose (*n* = 8)	8^a^	1.00	0	0.00	0	0.00	5^d^	0.63 (0.13–0.88)
Red deer (*n* = 28)	28^a^	1.00	0	0.00	0	0.00	20^e^	0.71 (0.46–0.82)
Roe deer (*n* = 7)	7^b^	1.00	0	0.00	0	0.00	7^f^	1.00
Wild boar (*n* = 34)	24^a^	0.71 (0.50–0.82)	0	0.00	0	0.00	0	0.00

*n*_*P*_ Number of positive animals,* IP* infection prevalence with 95% CI (95% CI are 95% bootstrapped, bias-corrected CI)

^a^42 *A. phagocytophilum*-positive samples from fallow deer, two from moose, 20 from red deer and seven from wild boar were sequenced; all were ecotype 1

^b^All *A. phagocytophilum*-positive samples from roe deer were sequenced; all were ecotype 2

^c^Eight *Babesia* spp.-positive samples from fallow deer were sequenced: two *B. capreoli*, two *B. divergens* and three *B. odocoilei-EU*

^d^Three *Babesia* spp.-positive samples from moose were sequencend: *B. odocoilei-EU*

^e^16 *Babesia* spp. positive-samples from red deer were sequenced: six *B. divergens*, three *B. odocoilei-EU*, one *B. venatorum* and six *B. divergens* and *B. venatorum*

^f^All *Babesia* spp.-positive samples from roe deer were sequenced: three *B. capreoli*, three *B. capreoli* and *B. venatorum* and one *B. capreoli*, *B. divergens* and *B. venatorum*, respectively

### Infection prevalence of tick-borne pathogens in questing ticks

We tested 811 questing *I. ricinus* nymphs and 84 adults for the presence of tick-borne pathogens. All investigated pathogens were present in the questing *I. ricinus* nymphs and adults at low prevalence rates (< 5%), except for *B. burgdorferi* (*s.l.*) and *A. phagocytophilum*, both of which occurred at higher prevalence rates (Table 4). The *Haemaphysalis punctata* male was negative for all investigated pathogens and was excluded from further analyses.

**Table 4:** Infection prevalence of tick-borne pathogens in questing *Ixodes ricinus* ticks

Life stage	*Anaplasma phagocytophilum*		*Borrelia burgdorferi* (*s.l.*)		*Borrelia miyamotoi*		*Babesia* spp.	
	*n*_P_	IP (95% CI)	*n*_P_	IP (95% CI)	*n*_P_	IP (95% CI)	*n*_P_	IP (95% CI)
Nymphs (*n* = 881)	36	0.04 (0.03–0.05)	136	0.15 (0.13–0.18)	8	0.01 (0.00–0.02)	8	0.01 (0.00–0.02)
Adults (*n* = 84)	8	0.10 (0.04–0.15)	16	0.19 (0.11–0.26)	1	0.01 (0.00–0.04)	3	0.04 (0.00–0.07)

*n*_P_ and IP (95% CI) are as defined in footnote of Table 3

### Contribution of ungulate species to the transmission of *Anaplasma phagocytophilum* and *Borrelia burgdorferi* (*s.l.*) through larvae

We included 285 ungulates that were checked for larvae and nymphs on at least both ears in the analyses. We found 24 animals that were infested with 59 larvae in total on the ears (Additional file 1: Table S3). A Šidák-adjusted Dunn-test showed that infestation prevalence of larvae differed between fallow deer and red deer (*P* = 0.011) and between fallow deer and wild boar (*P* < 0.001) (Table 2). The infestation intensity of larvae did not differ among the ungulate species (Kruskal–Wallis *χ*^2^ = 0.55; *P* = 0.76) (Table 2).

We tested 56 feeding larvae for the presence of tick-borne pathogens. Of these, 77% were positive for *A. phagocytophilum* and 5% for *B. burgdorferi* (*s.l.*) (Additional file 1: Table S6). Sequencing showed that *B. afzelli*, *B. burgdorferi* sensu stricto, *B. garinii* and *B. valaisiana* were present among the ticks (Additional file 1: Table S8). A Šidák-adjusted Dunn-test showed that there was a difference in *A. phagocytophilum* infection prevalence in feeding larvae from red deer and roe deer (*p* = 0.015) (Table 2). We found no difference in *B. burgdorferi* (*s.l.*) infection prevalence in feeding larvae among the ungulate species (Kruskal–Wallis test: *χ*^2^ = 2.38; *P* = 0.49) (Table 2).

The *A. phagocytophilum* infection intensity of engorged larvae, calculated with Eq. 2, was the highest for fallow deer and the lowest for moose, roe deer and wild boar, while red deer did not differ from any of the other ungulate species (Table 2; Additional file 1: Figure S3). The *B. burgdorferi* (*s.l.*) infection intensity of engorged larvae did not differ among the ungulate species (Table 2; Additional file 1: Figure S3).

### Contribution of ungulate species to the transmission of *Anaplasma phagocytophilum* and *Borrelia burgdorferi* (*s.l.*) through nymphs

Of the 285 checked individuals, we found 137 animals infested with 1308 nymphs in total on the ears (Additional file 1: Table S3). The selected models (Additional file 1: Table S7) suggested that fallow deer and roe deer had a higher infestation prevalence with nymphs than moose, red deer and wild boar. Of these latter three species, red deer had the highest infestation prevalence with nymphs and wild boar the lowest, while moose did not differ from red deer and wild boar (Table 2). Infestation intensity with nymphs did not differ among fallow deer, red deer and roe deer (Table 2). We excluded moose and wild boar for this parameter because of low sample sizes (Additional file 1: Table S3).

We tested 1309 feeding nymphs for the presence of tick-borne pathogens. Of these, 84% were positive for *A. phagocytophilum* and 5% for *B. burgdorferi* (*s.l.*) (Additional file 1: Table S6). In the models for *A. phagocytophilum* and *B. burgdorferi* (*s.l.*) infection prevalence in nymphs, we excluded moose and wild boar since there were only five and seven nymphs, respectively, tested from these species (Additional file 1: Table S6). Based on the selected models (Additional file 1: Tables S9, S10), there was no difference among fallow deer, red deer and roe deer in terms of the *A. phagocytophilum* and the *B. burgdorferi* (*s.l.*) infection prevalence in nymphs (Table 2).

We could not calculate the infection intensity for moose and wild boar since we did not obtain the infestation intensity due to a low number of animals infested. Among the other ungulate species, we did not find any difference in *A. phatocyophilum* or *B. burgdorferi* (*s.l.*) infection intensity of engorged nymphs (Table 2, Additional file 1: Figure S3).

### Transmission of *Anaplasma phagocytophilum* and *Borrelia burgdorferi* (*s.l.*) from ungulate host to ticks

The *A. phagocytophilum* infection prevalence was lower in questing nymphs than in feeding nymphs from fallow deer (*P* < 0.001), moose (*P* = 0.005), red deer (*P* < 0.001) and roe deer (*P* < 0.001), but not for wild boar (*P* = 0.038). The *B. burgdorferi* (*s.l.*) infection prevalence was higher in questing nymphs than in feeding nymphs from fallow deer (*P* < 0.001), red deer (*P* = 0.004) and roe deer (*P* < 0.001), but there was no difference for moose (*P* = 0.840) and wild boar (*P* = 0.703). We tested 1211 feeding females and 623 non-feeding males derived from ungulates for the presence of tick-borne pathogens and compared the infection prevalence with the infection prevalence in questing adults. Of the feeding females, 92% were positive for *A. phagocytophilum*, as were 76% of the males (Additional file 1: Table S6). The infection prevalence in questing adults was lower than that in feeding females from all ungulates (*P* < 0.001) and it was lower than the infection prevalence in non-feeding males from fallow deer (*P* < 0.001), moose (*P* < 0.001), red deer (*P* < 0.001) and roe deer (*P* < 0.001). There was no difference in infection prevalence between the questing adults and the non-feeding males from wild boar. We found that 8% of the feeding females and 14% of the non-feeding males were positive for *B. burgdorferi* (*s.l.*) (Additional file 1: Table S6). The infection prevalence was higher in questing adults than in feeding females from fallow deer (*P* = 0.023), moose (*P* = 0.024), red deer (*P* < 0.001) and roe deer (*P* < 0.001), while there was no difference for wild boar (*P* = 0.807). For all ungulate species there was no difference in infection prevalence between the non-feeding males and questing adults (Kruskal–Wallis test: *χ*^2^ = 6.75;* P* = 0.24).

### *Babesia* spp. and *Borrelia miyamotoi* in ticks collected from ungulates

Of the feeding larvae, 4% were positive for *Babesia* spp., as were 5% of the feeding nymphs, 13% of the feeding females and 6% of the non-feeding males (Additional file 1: Table S6). Among the positive ticks, we found *B. microti*, *B. capreoli*, *B. venatorum*, *B. divergens* and *B. odocoilei-EU* (Additional file 1: Table S11). The infection prevalence of *Babesia* spp. was higher in feeding nymphs than in questing nymphs for red deer (*P* < 0.001) and roe deer (*P* < 0.001), while there was no difference for the other ungulate species. The infection prevalence was higher in feeding females than in questing adults for red deer (*P* < 0.001) and roe deer (*P* = 0.009), but not for the other ungulate species. There was no difference in infection prevalence between the non-feeding males and questing adults for any of the ungulate species (Kruskal–Wallis test: *χ*^2^ = 7.30; *P* = 0.20) .

Furthermore, we found that 2% of feeding larvae, 2% of feeding nymphs, 1% of feeding females and 0.5% of non-feeding males were positive for *B. miyamotoi* (Additional file 1: Table S6). The infection prevalence of feeding nymphs, feeding females and non-feeding males was not different from the infection prevalence of questing nymphs and questing adults, respectively (all Kruskal–Wallis test: Nymphs, *χ*^2^ = 5.03; *P* = 0.41; Females, *χ*^2^ = 9.11; *P* = 0.10; Males, *χ*^2^ = 3.78; *P* value = 0.58).

## Discussion

In this study we determined the relative contribution of different ungulate species as propagation hosts by comparing the infestation prevalence of non-feeding males and the female tick burden. All deer species we studied had a similar female tick burden and infestation prevalence of non-feeding males and, thus, played a similar role as propagation host in the life-cycle of *I. ricinus* (Fig. 3). For wild boar, we could not calculate the female tick burden due to a low number of *I. ricinus*-infested individuals despite a relatively high number of sampled individuals. Based on this low number, and on the low infestation prevalence, we conclude that, in our study, the role of wild boar as propagation host is negligible (Fig. 3). The contribution of the ungulate species to the transmission of *A. phagocytophilum* and *B. burgdorferi* (*s.l.*) was determined by comparing the infection intensity in larvae and nymphs. The *A. phagocytophilum* infection intensity in larvae was higher in fallow deer and red deer than in the other studied ungulate species. In nymphs, it was similar for fallow deer, red deer and roe deer, but could not be determined for wild boar and moose due to the low number of individuals infested with nymphs. Due to the low infestation prevalence in wild boar, we conclude that the role of wild boar in the transmission of *A. phagocytophilum* is negligible compared to that of fallow deer, red deer and roe deer (Fig. 3). For moose, we cannot draw definitive conclusions since the number of sampled moose was too low. The *B. burgdorferi* (*s.l.*) infection intensity in larvae was similar among all studied ungulate species, as well as in nymphs for fallow deer, red deer and roe deer. Again, we could not determine the *B. burgdorferi* (*s.l.*) infection intensity for wild boar and moose. However, given the low prevalence rates we can conclude that the role of wild boar in *B. burgdorferi* transmission is negligible compared to fallow deer, red deer and roe deer (Fig. 3).

The infestation prevalence and intensity varied among the ungulate species in our study, but in general we found lower numbers than other studies previously conducted in Europe [8–10, 41, 42]. Since the questing tick densities in our area were also lower than in other European studies, we believe that the main reason for the lower infestation prevalence and intensity might be geographical. Aspects like climate, vegetation and general mammal density might be different in our study area than elsewhere in Europe and explain lower tick densities. However, another reason might be that we sampled ticks from hunted animals and were therefore restricted to the hunting season to obtain abundant ungulate samples. Hunting season, however, occurs towards the end of the tick season, while in other studies sampling occurred either during the peak season or throughout the season. The fact that we sampled late in the season could therefore also partly explain the relatively low infestation prevalence and intensity we found. For all ungulate species, we found a low larval tick burden, which has also been found in other studies in Europe [43–46]. We found the majority of nymphs attached to the ears of ungulates, while adults were mainly attached to the groin and axilla, which is in line with results of previous studies on roe deer [9, 15]. The aim of our study was to test if and how ungulate species identity matters in terms of the spread of tick-borne pathogens—and not to determine the absolute tick burden and infection prevalence of the ungulate species. We conclude that ungulate species does indeed matter for this part of Sweden and during the late season. This is an important finding and highlights that we should investigate whether the differences among ungulate species that we found hold for other areas in Europe and/or during other seasons. For example, the differences among species that we identified may be even more pronounced earlier in the season, when the density of questing ticks is higher.

Our findings provide initial support for our suggestion that behavioral and morphological traits might drive differences in the role of different ungulate species in the life-cycle of *I. ricinus*. The concentration of adult ticks in the groin and axilla of all species indicates that, although the access points for ticks on different ungulate species might differ [47], most adult ticks migrate to the groin and axilla, suggesting that host leg length could play a larger role in determining the tick burden of ungulates than body mass. This may explain why we found a relatively low tick burden on moose, which have particularly long legs [48]. Moreover, we found the highest infestation prevalence and intensity of nymphs on the ears of fallow deer, which supports our hypothesis that feeding type influences tick infestation rates, since fallow deer graze more than the other deer species [17]. Ticks, which are mostly questing on ground vegetation, will more easily access grazing ungulates* via* the ears (and the head) than species that more frequently browse vegetation strata higher up, such as moose [49].

For all ungulate species, the *A. phagocytophilum* infection prevalence we found in feeding ticks (0.76–1.00) was high relative to values reported in earlier studies (0.22–0.86) [11, 43, 50]. Furthermore, we found a higher infection prevalence in feeding ticks than in questing ticks. This suggests that ungulates are important transmission hosts for *A. phagocytophilum* in this part of Sweden, despite tick infestation being relatively low, and that the infection prevalence of ungulate hosts influences the infection prevalence in feeding ticks. Within Europe there is much variation in infection prevalence in ungulate hosts [14], which might explain why the infection prevalence in feeding ticks reported in other studies was lower. In our study, the transmission cycle of *A. phagocytophilum* was mainly between nymphs and females. This has been proposed [51], but had not been shown in a field study. Moreover, non-feeding males in our study were infected with *A. phagocytophilum* and this infection prevalence was higher than in questing adults. This finding may suggest that *A. phagocytophilum* alters tick behavior, causing them to select for ungulates, or that males actually become infected with *A. phagocytophilum* between the time of questing and when we collected them from the animals [52]. The latter might happen when a male briefly feeds on a host before finding a female to mate with, or males might feed on the females they are attached to during mating and become infected through the female. However, our data do not allow us to draw conclusions on exact transmission pathways and we encourage others to investigate these potential mechanisms in targeted studies. More generally, our interpretation of the differences in infection prevalence between questing ticks and feeding ticks has its limitations because we did not investigate the exact transmission dynamics of tick-borne pathogens. However, our results can be used to generate hypotheses on the role of different ungulate species in the transmission pathways of tick-borne pathogens, which should be further investigated in future research. In fact, this remains a major knowledge gap for the field of tick-borne pathogens in general.

For *B. burgdorferi* (*s.l.*) we found a low infection prevalence in feeding ticks for all ungulate species, reflecting results in other studies [9, 10, 41, 42, 53, 54]. This low infection prevalence in feeding ticks for *B. burgdorferi* (*s.l.*), combined with our finding that infection prevalence was lower in feeding ticks than in questing ticks, support the notion that ungulates do not transmit the bacterium and that there might even be a borreliacidal effect [9, 55]. However, we still found *B. burgdorferi* (*s.l.*) in engorged ticks, which has also been shown in other studies [9, 10, 41, 42, 54]. Although these observations contradict the borreliacidal effect, we cannot rule out that the *B. burgdorferi* (*s.l.*) we detected in ticks were not-infectious bacteria. The infection prevalence in feeding larvae was low but not zero, which might indicate some co-feeding transmission between feeding nymphs or females and feeding larvae. Co-feeding transmission of *B. burgdorferi* (*s.l.*) has not yet been identified in ungulates, but has been demonstrated in mice (reviewed in [56]).

Although we made every effort to collect a sufficient sample size, the sample size for several of our ungulate species was still quite limited (especially for moose and roe deer). This was, at least partly, due to the relatively low densities of these species in our study area [57] and the resulting low hunting quota. These low sample sizes might explain why some of the differences among the ungulate species in terms of female tick burden and infection intensity in larvae and nymphs were non-significant. The aim of our study was to compare different ungulate species and, therefore, we only draw conclusions on the relative contribution of the five studied ungulate species. To investigate the overall contribution of ungulates species, we should have included other host species in our study. Furthermore, we cannot draw any conclusions about the absolute tick burden and infection prevalence for each ungulate species. Our initial results, which indicate that ungulate species identity matters, do strongly suggest that future research should quantify the absolute contribution of different ungulate species to the dynamics of tick-borne pathogens. Such work should focus on essential parameters, such as exact transmission pathways and persistent infection, which we did not include in our study. Such studies have been performed in rodents (e.g., [28]), but not yet in ungulates.

Our study included only *I. ricinus* and included tick-borne pathogens for which this tick species is the main vector in Europe [58]. However, we do suggest that similar results may hold for a broader collection of tick species and their pathogens. The main pathogens investigated in our study, *B. burgdorferi* (*s.l.*) and *A. phagocytophilum*, are globally not limited to the tick species *I. ricinus* [58], and it is likely that the morphological and behavioral differences among the ungulate species also influence their ability to feed other tick species.

## Conclusion

Despite our relatively low sample sizes, we found support for our main hypothesis that the different ungulate species may play a different role in the propagation of ticks and the transmission cycles of tick-borne pathogens. For example, in our system wild boar played a small role as propagation host, and fallow deer seemed to play a stronger role in the transmission cycle of *A. phagocytophilum* relative to the other deer species. Given our small sample sizes, we urge others to challenge and confirm our preliminary findings and invest more effort in comparing the role of different sympatric ungulate species in the spread of tick-borne pathogens in other systems and during other seasons. If our results hold, this means that ungulate management, as a tool to mitigate zoonotic disease risk, should not treat ungulates as one black box. Rather, such management should take the potentially different roles of different species as propagation hosts and in pathogen transmission into account and acknowledge that these roles may vary depending on the target pathogen. Our initial results suggest that choices in ungulate management, for example targeting specific ungulate species differently, could markedly influence the impact of the strategy on the abundance of infected questing ticks.

## Supplementary Information

**Additional file 1: Table S1.** Number of individuals per ungulate species included in the study. **Table S2.** Proportion (%) of ticks found on different body parts of the five studied ungulate species. **Table S3.** Summary of feeding larvae and feeding nymphs on ears, feeding females on groin and axilla and non-feeding males on complete carcasses on five ungulate species. **Table S4.** Standardized model estimates with 95% confidence intervals for the analysis of infestation prevalence with non-feeding males. **Table S5.** Standardized model estimates with 95% confidence intervals for the analysis of infestation prevalence (A) and intensity (B) with feeding females. **Table S6.** Infection prevalence of tick-borne pathogens in feeding *Ixodes ricinus* ticks from five studied ungulate species. **Table S7.** Standardized model estimates with 95% confidence intervals for the analysis of infestation prevalence (A) and intensity (B) with feeding nymphs. **Table S8.** Sequencing results from *Borrelia burgdorferi* (*s.l.*) positive ticks collected from ungulates. **Table S9.** Standardized model estimates with 95% confidence intervals for the analysis of the infection prevalence of *Anaplasma phagocytophilum* in feeding nymphs. **Table S10.** Standardized model estimates with 95% confidence intervals for the analysis of the infection prevalence of *Borrelia burgdorferi* (*s.l.*) in feeding nymphs. **Table S11.** Sequencing results from *Babesia* ssp. positive ticks collected from ungulates. **Figure S1.** Map of Sweden with the study area in green. **Figure S2.** Larval (**A**), nymphal (**B**) and female (**C**) tick burden on the studied ungulate species. Tick burden, as calculated by formula 1, is given with 84% bootstrapped, bias-corrected, confidence intervals to show differences among ungulate species with a significance with an alpha value of 0.05. **Figure S3.** Infection intensity in larvae and nymphs from the studied ungulate species. Infection intensity, as calculated by formula 2, is given with 84% bootstrapped, bias-corrected, confidence intervals to show differences among ungulate species with a significance with an alpha value of 0.05. The four graphs show the *Anaplasma phagocytophilum* infection intensity in larvae (**A**) and nymphs (**B**) and the *Borrelia burgdorferi* (*s.l.*) infection intensity in larvae (**C**) and nymphs (**D**).

**Additional file 2: **Raw dataset.

## Data Availability

All data generated or analyzed during this study are included in this published article and its Additional files.
