# Supplementary material for: Wild ungulate species differ in their contribution to the transmission of Ixodes ricinus-borne pathogens
Source: Parasit Vectors. 2021 Jul 10;14:360. doi: 10.1186/s13071-021-04860-w (PMC8272276; doi:10.1186/s13071-021-04860-w)
Supplement: Supplementary file 1 — Additional file 1: Table S1. Number of individuals per ungulate species included in the study. Table S2. Proportion (%) of ticks found on different body parts of the five studied ungulate species. Table S3. Summary of feeding larvae and feeding nymphs on ears, feeding females on groin and axilla and non-feeding males on complete carcasses on five ungulate species. Table S4. Standardized model estimates with 95% confidence intervals for the analysis of infestation prevalence with non-feeding males. Table S5. Standardized model estimates with 95% confidence intervals for the analysis of infestation prevalence (A) and intensity (B) with feeding females. Table S6. Infection prevalence of tick-borne pathogens in feeding Ixodes ricinus ticks from five studied ungulate species. Table S7. Standardized model estimates with 95% confidence intervals for the analysis of infestation prevalence (A) and intensity (B) with feeding nymphs. Table S8. Sequencing results from Borrelia burgdorferi (s.l.) positive ticks collected from ungulates. Table S9. Standardized model estimates with 95% confidence intervals for the analysis of the infection prevalence of Anaplasma phagocytophilum in feeding nymphs. Table S10. Standardized model estimates with 95% confidence intervals for the analysis of the infection prevalence of Borrelia burgdorferi (s.l.) in feeding nymphs. Table S11. Sequencing results from Babesia ssp. positive ticks collected from ungulates. Figure S1. Map of Sweden with the study area in green. Figure S2. Larval (A), nymphal (B) and female (C) tick burden on the studied ungulate species. Tick burden, as calculated by formula 1, is given with 84% bootstrapped, bias-corrected, confidence intervals to show differences among ungulate species with a significance with an alpha value of 0.05. Figure S3. Infection intensity in larvae and nymphs from the studied ungulate species. Infection intensity, as calculated by formula 2, is given with 84% bootstrapped, bias-corrected, confiden [file 13071_2021_4860_MOESM1_ESM.pdf]

Wild ungulate species differ in their contribution to the transmission of Ixodes ricinus-borne pathogens.

Nannet D. Fabri, Hein Sprong, Tim R. Hofmeester, Hans Heesterbeek, Björn F. Donnars, Fredrik Widemo, Frauke Ecke and Joris P.G.M. Cromsigt

Additional file 1: Additional tables and figures

Table S1. Number of individuals per ungulate species included in the study.

	Fallow deer	Moose	Red deer	Roe deer	Wild boar
Individuals where a part of the carcass was checked for ticks and no spleen sample was obtained	15	4	6	3	1
Individuals where the whole carcass was checked for ticks and no spleen sample was obtained	52	3	27	20	52
Individuals where only a spleen sample was obtained	16	0	2	0	1
Individuals where a part of the carcass was checked for ticks and a spleen sample was obtained	7	1	3	1	3
Individuals where the whole carcass was checked for ticks and a spleen sample was obtained	41	7	23	6	30
Total number of individuals	131	15	61	30	87

Table S2. Proportion (%) of ticks found on different body parts of the five studied ungulate species.

	Feeding larvae		Feeding nymphs		Feeding females	
**Fallow deer**	Larvae (n=42)		Nymphs (n=1015)		Females (n=464)	
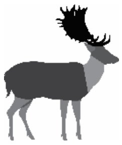	**Ears**	**97.6**	**Ears**	**98.8**	Ears	0.7
	Head	0	Head	0.1	Head	0.2
	Neck	0	Neck	0	Neck	0
	Front leg	0	Front leg	0	Front leg	0
	Axilla	0	Axilla	0.6	Axilla	8.2
	Hind leg	0	Hind leg	0	Hind leg	0.2
n = 93	Groin	0	Groin	0.2	**Groin**	**88.1**
infested = 88	Other	0	Other	0	Other	1.1
	Unknown	2.4	Unknown	0.2	Unknown	1.5
	Larvae (n=0)		Nymphs (n=2)		Females (n=26)	
**Moose**			**Ears**	**100**	Ears	15.4
			Head	0	Head	0
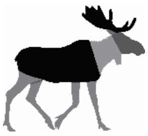			Neck	0	Neck	3.8
			Front leg	0	Front leg	0
			Axilla	0	Axilla	15.4
			Hind leg	0	Hind leg	0
n = 10			Groin	0	**Groin**	**65.4**
infested = 10			Other	0	Other	0
			Unknown	0	Unknown	0
	Larvae (n=3)		Nymphs (n=88)		Females (n=331)	
**Red deer**	**Ears**	**100**	**Ears**	**97.8**	Ears	1.5
	Head	0	Head	1.1	Head	0
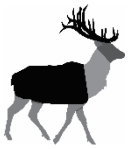	Neck	0	Neck	0	Neck	0.3
	Front leg	0	Front leg	0	Front leg	0
	Axilla	0	Axilla	0	Axilla	7.6
	Hind leg	0	Hind leg	0	Hind leg	0
n = 50	Groin	0	Groin	0	**Groin**	**89.1**
infested = 47	Other	0	Other	0	Other	0.9
	Unknown	0	Unknown	1.1	Unknown	0.6
	Larvae (n=7)		Nymphs (n=122)		Females (n=132)	
**Roe deer**	**Ears**	**100**	**Ears**	**99.2**	Ears	0.8
	Head	0	Head	0	Head	0
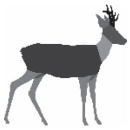	Neck	0	Neck	0	Neck	0
	Front leg	0	Front leg	0	Front leg	0
	Axilla	0	Axilla	0	Axilla	11.4
	Hind leg	0	Hind leg	0.8	Hind leg	2.3
n = 26	Groin	0	Groin	0	**Groin**	**85.6**
infested = 24	Other	0	Other	0	Other	0
	Unknown	0	Unknown	0	Unknown	0
	Larvae (n=0)		Nymphs (n=6)		Females (n=13)	
			**Ears**	**100**	Ears	7.7
**Wild boar**			Head	0	Head	0
			Neck	0	Neck	0
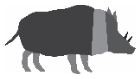			Front leg	0	Front leg	0
			Axilla	0	Axilla	7.7
n = 82			Hind leg	0	Hind leg	0
infested = 13			Groin	0	**Groin**	**53.8**
			Other	0	Other	30.8
			Unknown	0	Unknown	0

Percentages higher than 50% are given in bold and number of samples are given in parentheses. Only individuals of which the entire carcass was checked for ticks were included. Silhouettes by Sander Vink.

Table S3. Summary of feeding larvae and feeding nymphs on ears, feeding females on groin and axilla and non-feeding males on complete carcasses on five ungulate species.

	Ears			*Groin and axilla*		*Complete carcass*	
	n_e	**Feeding larvae**	**Feeding nymphs**	n_{ga}	**Feeding females**	n_c	**Non-feeding males**
**Fallow deer**	104	Larvae (n=47)<br>Infested individuals = 19<br>Mean D_L = 0.45 (0.25-0.84)<br>Mean P_L = 0.18 (0.11-0.26)<br>Mean I_L = 2.47 (1.79-3.84)	Nymphs (n=1 071)<br>Infested individuals = 88<br>Mean D_N = 10.30 (8.16-13.71)<br>Mean P_N = 0.85 (0.76-0.90)<br>Mean I_N = 12.17 (10.13-16.57)	115	Females (n=514)<br>Infested individuals = 99<br>Mean D_F = 4.47 (3.70-5.36)<br>Mean P_F = 0.86 (0.77-0.91)<br>Mean I_F = 5.19 (4.47-6.19)	93	Males (n=108)<br>Infested individuals = 44<br>Mean D_M = 1.16 (0.77-1.87)<br>Mean P_M = 0.47 (0.37-0.57)<br>Mean I_M = 2.45 (1.80-3.94)
**Moose**	15	Larvae (n=0)<br>Infested individuals = 0<br>Mean D_L = 0.00<br>Mean P_L = 0.00<br>Mean I_L = 0.00	Nymphs (n=4)<br>Infested individuals = 3<br>Mean D_N = 0.27 (0.00-0.60)<br>Mean P_N = 0.20 (0.00-0.40)<br>Mean I_N = 1.33 (1.00-1.67)	14	Females (n=33)<br>Infested individuals = 13<br>Mean D_F = 2.36 (1.44-3.57)<br>Mean P_F = 0.93 (0.64-1.00)<br>Mean I_F = 2.54 (1.62-4.01)	10	Males (n=66)<br>Infested individuals = 10<br>Mean D_M = 6.60 (4.20-8.80)<br>Mean P_M = 1.00[b]<br>Mean I_M = 6.60 (4.14-8.90)
**Red deer**	52	Larvae (n=5)<br>Infested individuals = 2<br>Mean D_L = 0.10 (0.00-0.29)<br>Mean P_L = 0.04 (0.00-0.10)<br>Mean I_L = 2.50 (2.00-2.50)	Nymphs (n=97)<br>Infested individuals = 18<br>Mean D_N = 1.87 (0.84-3.68)<br>Mean P_N = 0.35 (0.21-0.46)<br>Mean I_N = 5.39 (2.72-10.04)	58	Females (n=384)<br>Infested individuals = 50<br>Mean D_F = 6.62 (5.17-8.61)<br>Mean P_F = 0.86 (0.71-0.91)<br>Mean I_F = 7.68 (6.00-9.82)	50	Males (n=269)<br>Infested individuals = 44<br>Mean D_M = 5.38 (3.92-7.29)<br>Mean P_M = 0.88 (0.76-0.94)<br>Mean I_M = 6.11 (4.59-8.16)
**Roe deer**	29	Larvae (n=7)<br>Infested individuals = 3<br>Mean D_L = 0.24 (0.03-0.72)<br>Mean P_L = 0.10 (0.00-0.21)<br>Mean I_L = 2.33 (1.00-3.33)	Nymphs (n=129)<br>Infested individuals = 21<br>Mean D_N = 4.45 (2.90-7.39)<br>Mean P_N = 0.72 (0.52-0.83)<br>Mean I_N = 6.14 (4.03-9.80)	29	Females (n=133)<br>Infested individuals = 25<br>Mean D_F = 4.59 (3.28-6.31)<br>Mean P_F = 0.86 (0.62-0.93)<br>Mean I_F = 5.32 (4.03-6.88)	26	Males (n=68)<br>Infested individuals = 19<br>Mean D_M = 2.62 (1.75-3.76)<br>Mean P_M = 0.73 (0.42-0.85)<br>Mean I_M = 3.58 (2.68-4.79)
**Wild boar**	85	Larvae (n=0)<br>Infested individuals = 0<br>Mean D_L = 0.00<br>Mean P_L = 0.00<br>Mean I_L = 0.00	Nymphs (n=7)<br>Infested individuals = 7<br>Mean D_N = 0.08 (0.02-0.14)<br>Mean P_N = 0.08 (0.02-0.14)<br>Mean I_N = 1.00[a]	86	Females (n=8)<br>Infested individuals = 5<br>Mean D_F = 0.09 (0.03-0.22)<br>Mean P_F = 0.06 (0.01-0.12)<br>Mean I_F = 1.60 (1.00-2.20)	82	Males (n=4)<br>Infested individuals = 2<br>Mean D_M = 0.05 (0.00-0.20)<br>Mean P_M = 0.02 (0.00-0.06)<br>Mean I_M = 2.00 (1.00-3.00)

n_e = number of animals where at least the ears were checked for ticks, n_{ga} = number of animals where at least the groin and axilla were checked for ticks, n_c = number of animals where the complete carcass was checked for ticks, mean D = mean infestation density, mean P = mean infestation prevalence, mean I = mean infestation intensity. 95% bootstrapped, bias-corrected, confidence intervals in brackets. [a] All infested animals were infested with the same amount of ticks, thus a 95% CI for mean infestation intensity could not be calculated. [b] All animals were infested, thus a 95% CI for mean prevalence of infestation could not be calculated.

Table S4. Standardized model estimates with 95% confidence intervals for the analysis of infestation prevalence with non-feeding males. Models presented are the best performing hierarchical GLMMs with a binomial distribution. The model selected for the analyses is bold.

	Model 1			Model 2			Model 3			**Model 4**		
	Est.	95% CI		Est.	95% CI		Est.	95% CI		**Est.**	**95% CI**	
		Low.	Upp.		Low.	Upp.		Low.	Upp.		**Low.**	**Upp.**
Moose[a]	23.58	-47 495.02	47 542.19	26.53	-20 8162.8	20 8215.8	24.47	-75 763.85	75 812.80	**30.86**	**-20 124.57**	**20 125.18**
Red deer[a]	3.49	2.00	4.97	3.47	1.99	4.96	3.49	2.00	4.98	**3.30**	**1.96**	**4.64**
Roe deer[a]	1.35	0.15	2.55	1.37	0.16	2.59	1.35	0.15	2.55	**1.23**	**0.07**	**2.39**
Wild boar[a]	-3.70	-5.19	-2.21	-3.72	-5.21	-2.22	-3.69	-5.18	-2.19	**-3.73**	**-5.22**	**-2.24**
October 2019[b]	-0.17	-1.17	0.84	-0.15	-1.17	0.86	-0.18	-1.19	0.83	**-0.24**	**-1.11**	**0.64**
November 2019 [b]	-2.90	-4.35	-1.45	-2.89	-4.34	-1.44	-2.92	-4.38	-1.46	**-2.65**	**-3.93**	**-1.36**
Freshness	-0.08	-0.53	0.37	-0.08	0.53	0.37	-0.08	-0.53	0.37	**—**	**—**	**—**
Male[c]	—	—	—	—	—	—	-0.10	-0.68	0.87	**—**	**—**	**—**
Young[d]	—	—	—	0.11	-0.66	0.87	—	—	—	**0.04**	**-0.72**	**0.79**
∆AIC	0.00			2.10			2.12			**3.80**		

[a] Standardized correlation coefficients as compared to zero for fallow deer.

[b] Standardized correlation coefficients as compared to zero for October 2018.

[c] Standardized correlation coefficient for males as compared to zero for females.

[d] Standardized correlation coefficient for young as compared to zero for adults.

— Parameter was not included in the model.

Table S5. Standardized model estimates with 95% confidence intervals for the analysis of infestation prevalence (A) and intensity (B) with feeding females. Models presented are the best performing hierarchical GLMMs with a binomial distribution for infestation prevalence and with a truncated negative binomial distribution for infestation intensity. The models selected for the analyses are bold.

A	Model 1			Model 2			Model 3			**Model 4**			Model 5			Model 6		
	Est.	95% CI		Est.	95% CI		Est.	95% CI		**Est.**	**95% CI**		Est.	95% CI		Est.	95% CI	
		Low.	Upp.		Low.	Upp.		Low.	Upp.		**Low.**	**Upp.**		Low.	Upp.		Low.	Upp.
Moose[a]	1.04	-1.38	3.47	0.83	-1.65	3.30	1.04	-1.38	3.47	**1.11**	**-1.27**	**3.49**	0.82	-1.65	3.30	0.90	-1.50	3.30
Red deer[a]	0.66	-0.67	1.99	0.60	-0.78	1.98	0.65	-0.67	1.98	**0.78**	**-0.50**	**2.06**	0.60	-0.78	1.98	0.74	-0.59	2.08
Roe deer[a]	0.34	-1.20	1.88	0.27	-1.30	1.84	0.38	-1.23	1.98	**0.30**	**-1.24**	**1.85**	0.25	-1.39	1.89	0.24	-1.33	1.81
Wild boar[a]	-6.22	-8.05	-4.39	-6.32	-8.20	-4.43	-6.25	-8.11	-4.38	**-6.25**	**-8.03**	**-4.46**	-6.31	-8.20	-4.42	-6.33	-8.17	-4.48
October 2019[b]	0.22	-1.36	1.81	0.21	-1.40	1.82	0.23	-1.36	1.82	**-0.03**	**-1.50**	**1.44**	0.21	-1.40	1.81	-0.03	-1.54	1.47
November 2019 [b]	-2.65	-4.16	-1.14	-2.70	-4.26	-1.14	-2.64	-4.16	-1.13	**-2.67**	**-4.19**	**-1.16**	-2.70	-4.26	-1.15	-2.75	-4.33	-1.17
Freshness	-0.30	-1.00	0.39	-0.31	-1.04	0.41	-0.31	-1.00	0.39	**—**	**—**	**—**	-0.31	-1.04	0.41	—	—	—
Male[c]	—	—	—	0.44	-0.55	1.44	—	—	—	**—**	**—**	**—**	0.45	-0.58	1.48	0.57	-0.41	1.54
Young[d]	—	—	—	—	—	—	0.08	-0.89	1.05	**—**	**—**	**—**	-0.04	-1.06	0.97	—	—	—
ΔAIC	0.00			1.36			2.12			**3.19**			3.52			3.97		

B	**Model 1**			Model 2		
	**Est.**	**95% CI**		Est.	95% CI	
		**Low.**	**Upp.**		Low.	Upp.
Moose[a]	**-1.00**	**-2.02**	**0.02**	-0.99	-1.99	$-0.28\cdot10^{-3}$
Red deer[a]	**0.35**	**-0.07**	**0.77**	0.34	-0.08	0.75
Roe deer[a]	**0.01**	**-0.40**	**0.42**	0.01	-0.40	0.41
Freshness	**-0.05**	**-0.27**	**0.17**	-0.06	-0.28	0.16
Male[c]	**—**	**—**	**—**	0.07	-0.20	0.34
Young[d]	**-0.42**	**-0.68**	**-0.16**	-0.44	-0.71	-0.17
ΔAIC	**0.00**			1.96		

[a] Standardized correlation coefficients as compared to zero for fallow deer.
[b] Standardized correlation coefficients as compared to zero for October 2018.
[c] Standardized correlation coefficient for males as compared to zero for females.
[d] Standardized correlation coefficient for young as compared to zero for adults.
— Parameter was not included in the model.

Table S6. Infection prevalence of tick-borne pathogens in feeding *Ixodes ricinus* ticks from five studied ungulate species.

				Anaplasma phagocytophilum[a]		*Borrelia burgdorferi* s.l.[b]		*Borrelia miyamotoi*		*Babesia* spp.[c]	
		n_t	n_h	n_P	IP (95% CI)	n_P	IP (95% CI)	n_P	IP (95% CI)	n_P	IP (95% CI)
**Fallow deer**	Feeding larvae	48	22	38	0.79 (0.63-0.88)	2	0.04 (0.00-0.10)	1	0.02 (0.00-0.06)	1	0.02 (0.00-0.06)
	Feeding nymphs	1067	89	916	0.86 (0.84-0.88)	50	0.05 (0.03-0.06)	20	0.02 (0.01-0.03)	30	0.03 (0.02-0.04)
	Feeding females	551	99	495	0.90 (0.87-0.92)	52	0.09 (0.07-0.12)	5	0.01 (0.00-0.02)	42	0.08 (0.05-0.10)
	Non-feeding males	114	49	95	0.83 (0,75-0.89)	21	0.18 (0.11-0.26)	0	0.00	3	0.03 (0.00-0.06)
**Moose**	Feeding larvae	1	1	0	0.00	0	0.00	0	0.00	0	0.00
	Feeding nymphs	5	4	4	0.80 (0.00-1.00)	0	0.00	0	0.00	0	0.00
	Feeding females	48	14	34	0.72 (0.55-0.81)	2	0.04 (0.00-0.11)	2	0.04 (0.00-0.11)	4	0.09 (0.02-0.15)
	Non-feeding males	133	15	84	0.63 (0.53-0.70)	12	0.09 (0.04-0.14)	0	0.00	8	0.06 (0.02-0.10)
**Red deer**	Feeding larvae	5	2	5	1.00	1	0.20 (0.00-0.40)	0	0.00	1	0.20 (0.00-0.40)
	Feeding nymphs	100	20	93	0.93 (0.85-0.96)	5	0.05 (0.01-0.09)	0	0.00	9	0.09 (0.04-0.14)
	Feeding females	445	46	434	0.98 (0.96-0.99)	26	0.06 (0.04-0.09)	1	0.00 (0.00-0.01)	83	0.19 (0.15-0.22)
	Non-feeding males	286	48	232	0.81 (0.76-0.85)	44	0.15 (0.11-0.19)	1	0.00 (0.00-0.01)	18	0.06 (0.04-0.09)
**Roe deer**	Feeding larvae	2	2	0	0.00	0	0.00	0	0.00	0	0.00
	Feeding nymphs	130	22	85	0.66 (0.55-0.73)	6	0.05 (0.02-0.09)	2	0.02 (0.00-0.04)	32	0.25 (0.18-0.32)
	Feeding females	155	26	140	0.90 (0.85-0.94)	10	0.06 (0.02-0.10)	2	0.01 (0.00-0.03)	28	0.18 (0.12-0.24)
	Non-feeding males	75	22	59	0.79 (0.67-0.87)	10	0.13 (0.05-0.21)	1	0.01 (0.00-0.04)	8	0.11 (0.04-0.19)
**Wild boar**	Feeding larvae	0	0	-	-	-	-	-	-	-	-
	Feeding nymphs	7	7	4	0.57 (0.00-0.71)	0	0.00	0	0.00	0	0.00
	Feeding females	12	7	11	0.92 (0.66-0.92)	1	0.08 (0.00-0.25)	0	0.00	1	0.08 (0.00-0.25)
	Non-feeding males	15	3	5	0.33 (0.07-0.53)	1	0.07 (0.00-0.20)	0	0.00	0	0.00

n_t = number of tested ticks, n_h = number of animals the tested ticks came from, n_P = number of ticks positive, IP = infection prevalence with 95% confidence interval in parentheses. The 95% confidence intervals are 95% bootstrapped, bias-corrected, confidence intervals.

[a] Ten positive non-feeding males from fallow deer, five from moose and nine from red deer were sequenced as ectoype 1. One from moose, two from red deer and five from roe deer were sequenced as ecotype 2.

[b] For sequencing results see Table S8

[c] For sequencing results see table S11

Table S7. Standardized model estimates with 95% confidence intervals for the analysis of infestation prevalence (A) and intensity (B) with feeding nymphs. Models presented are the best performing hierarchical GLMMs with a binomial distribution for infestation prevalence and with a truncated negative binomial distribution for infestation intensity. The models selected for the analyses are bold.

A	Model 1			**Model 2**		
	Est.	95% CI Low.	95% CI Upp.	**Est.**	**95% CI Low.**	**95% CI Upp.**
Moose[a]	-3.98	-5.62	-2.35	**-3.94**	**-5.55**	**-2.33**
Red deer[a]	-2.88	-3.89	-1.87	**-2.83**	**-3.83**	**-1.84**
Roe deer[a]	-0.36	-1.57	0.84	**-0.66**	**-1.80**	**0.49**
Wild boar[a]	-5.39	-6.66	-4.12	**-5.09**	**-6.28**	**-3.90**
October 2019[b]	-0.88	-1.88	0.12	**-0.92**	**-1.91**	**0.06**
November 2019 [b]	-2.23	-3.36	-1.10	**-2.22**	**-3.33**	**-1.11**
Freshness	-0.32	-0.77	0.14	**-0.30**	**-0.75**	**0.16**
Male[c]	-1.14	-1.94	-0.33	**-0.92**	**-1.68**	**-0.16**
Young[d]	0.89	0.07	1.70	—	—	—
ΔAIC	0.00			**2.59**		

[a] Standardized correlation coefficients as compared to zero for fallow deer.
[b] Standardized correlation coefficients as compared to zero for October 2018.
[c] Standardized correlation coefficient for males as compared to zero for females.
[d] Standardized correlation coefficient for young as compared to zero for adults.
— Parameter was not included in the model.

B	**Model 1**			Model 2			Model 3			Model 4		
	**Est.**	**95% CI Low.**	**95% CI Upp.**	Est.	95% CI Low.	95% CI Upp.	Est.	95% CI Low.	95% CI Upp.	Est.	95% CI Low.	95% CI Upp.
Red deer[a]	**-1.46**	**-2.83**	**-0.08**	-1.35	-2.65	-0.05	-1.43	-2.73	-0.14	-1.35	-2.65	-0.05
Roe deer[a]	**-0.81**	**-1.63**	**0.01**	-0.70	-1.50	0.10	-0.79	-1.59	0.01	-0.70	-1.50	0.10
Freshness	**0.01**	**-0.29**	**0.30**	0.01	-0.28	0.31	0.01	-0.29	0.31	0.01	-0.28	0.31
Male[c]	**—**	**—**	**—**	—	—	—	0.09	-0.35	0.53	-0.01	-0.45	0.44
Young[d]	**—**	**—**	**—**	0.26	-0.13	0.64	—	—	—	0.26	-0.15	0.66
ΔAIC	**0.00**			0.55			2.09			2.84		

Table S8. Sequencing results from *Borrelia burgdorferi* s.l. positive ticks collected from ungulates.

		Borrelia afzelli	***Borrelia burgdorferi* s.s.**	***Borrelia garinii***	***Borrelia valaisiana***	**Not sequenced**
**Fallow deer**	Feeding larvae	2	-	-	-	-
	Feeding nymphs	7	1	9	2	31
	Feeding females	5	1	3	-	43
	Non-feeding males	5	-	2	-	14
**Moose**	Feeding females	-	-	-	-	2
	Non-feeding males	1	-	-	-	11
**Red deer**	Feeding larvae	-	-	-	-	1
	Feeding nymphs	2	-	-	-	3
	Feeding females	2	1	-	-	23
	Non-feeding males	9	-	4	-	31
**Roe deer**	Feeding nymphs	5	-	-	-	1
	Feeding females	3	-	-	-	7
	Non-feeding males	1	1	-	-	8
**Wild boar**	Feeding females	-	-	-	-	1
	Non-feeding males	-	-	-	-	1

- No positive tick samples/No tick samples sequenced

Table S9. Standardized model estimates with 95% confidence intervals for the analysis of the infection prevalence of *Anaplasma phagocytophilum* in feeding nymphs.

Models presented are the best performing hierarchical GLMMs with a binomial distribution. The model selected for the analyses is bold.

	**Model 1**			Model 2		
	**Est.**	**95% CI**		Est.	95% CI	
		**Low.**	**Upp.**		Low.	Upp.
Red deer[a]	**0.01**	**-1.34**	**1.36**	$0.17*10^{-2}$	-1.35	1.35
Roe deer[a]	**-0.70**	**-1.67**	**0.27**	-0.72	-1.68	0.25
Male[b]	**—**	**—**	**—**	0.15	-0.62	0.92
Young[c]	**1.10**	**0.41**	**1.78**	1.04	0.31	1.77
∆AIC	**0.00**			1.87		

[a] Standardized correlation coefficients as compared to zero for fallow deer.

[b] Standardized correlation coefficient for males as compared to zero for females.

[c] Standardized correlation coefficient for young as compared to zero for adults.

— Parameter was not included in the model.

Table S10. Standardized model estimates with 95% confidence intervals for the analysis of the infection prevalence of *Borrelia burgdorferi* sensu lato in feeding nymphs.
Models presented are the best performing hierarchical GLMMs with a binomial distribution. The model selected for the analyses is bold.

	Model 1			Model 2			**Model 3**		
	Est.	95% CI		Est.	95% CI		**Est.**	**95% CI**	
		Low.	Upp.		Low.	Upp.		**Low.**	**Upp.**
Red deer[a]	-0.11	-1.05	0.84	$-0.49*10^{-2}$	-1.08	1.07	**0.14**	**-0.98**	**1.26**
Roe deer[a]	0.01	-0.78	0.97	0.18	-0.81	1.17	**$0.23*10^{-2}$**	**-1.00**	**1.01**
Male[b]	—	—	—	-0.18	-0.84	0.48	—	—	—
Young[c]	0.89	0.30	1.47	0.93	0.23	1.63	—	—	—
ΔAIC	0.00			0.09			**2.89**		

[a] Standardized correlation coefficients as compared to zero for fallow deer.
[b] Standardized correlation coefficient for males as compared to zero for females.
[c] Standardized correlation coefficient for young as compared to zero for adults.
— Parameter was not included in the model.

Table S11. Sequencing results from *Babesia* ssp. positive ticks collected from ungulates.

		Babesia microti	***Babesia capreoli***	***Babesia venatorum***	***Babesia divergens***	***Babesia odocoilei-EU***	**Not sequenced**
**Fallow deer**	Feeding larvae	1	-	-	-	-	-
	Feeding nymphs	15	1	5	-	-	9
	Feeding females	27	1	4	-	-	10
	Non-feeding males	2	-	1	-	-	-
**Moose**	Feeding females	1	-	-	-	-	3
	Non-feeding males	3	-	-	-	-	5
**Red deer**	Feeding larvae	-	-	-	-	-	1
	Feeding nymphs	-	3[a]	2[a]	1[a]	-	5
	Feeding females	15	1[b]	13	1	2[b]	52
	Non-feeding males	6	-	3	-	-	9
**Roe deer**	Feeding nymphs	1	4[c]	2[c]	-	-	26
	Feeding females	6	6	-	-	-	16
	Non-feeding males	4	-	1	-	-	3
**Wild boar**	Feeding females	-	-	1	-	-	-

[a] This includes one nymph that was positive for *B. capreoli, B. venatorum* and *B. divergens*
[b] This includes on female that was positive for *B. capreoli* and *B. odocoilei-EU*
[c] This includes one nymph that was positive for *B. capreoli* and *B. venatorum*

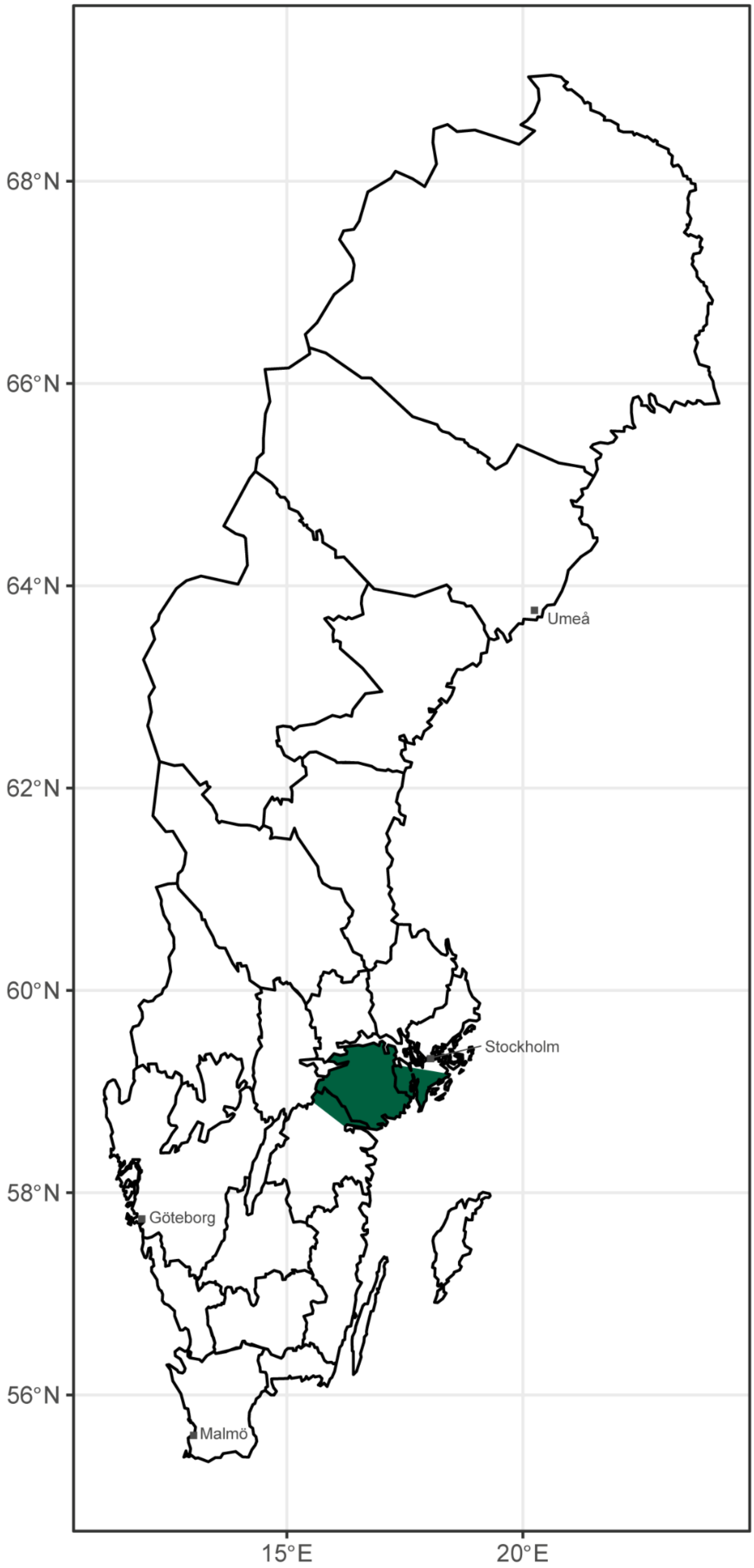

Figure S1. Map of Sweden with the study area in green.

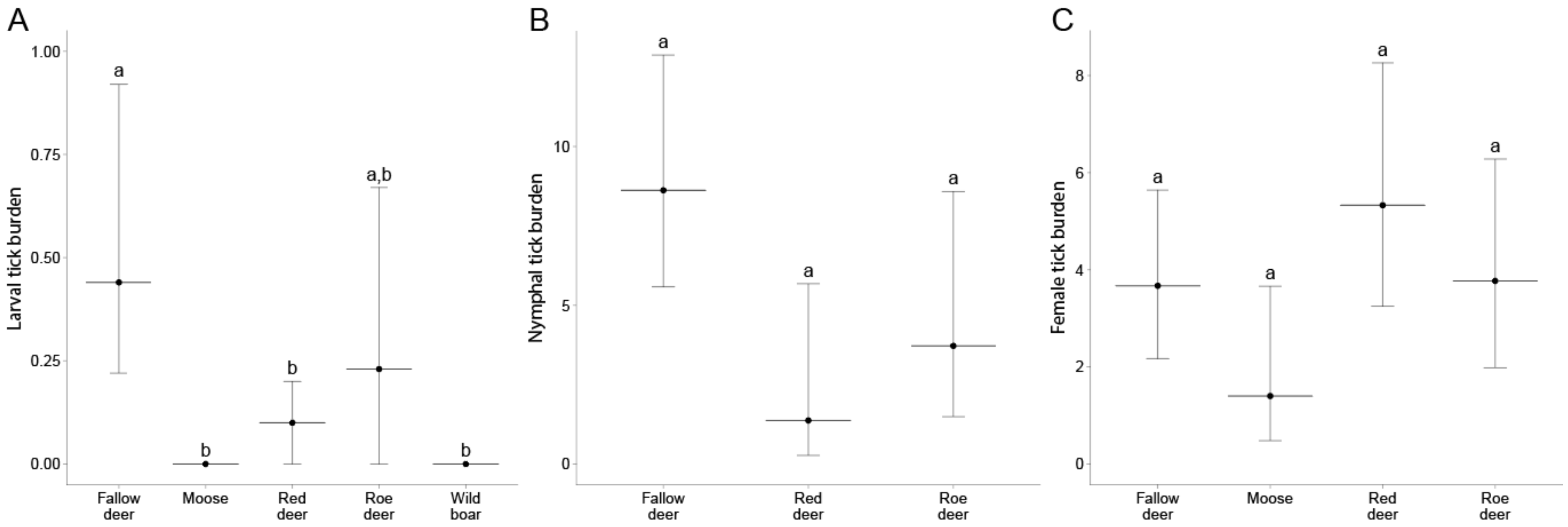

Figure S2. Larval (A), Nymphal (B) and Female (C) tick burden on the studied ungulate species. Tick burden, as calculated by formula 1, is given with 84% bootstrapped, bias-corrected, confidence intervals to show differences among ungulate species with a significance with an alpha value of 0.05.

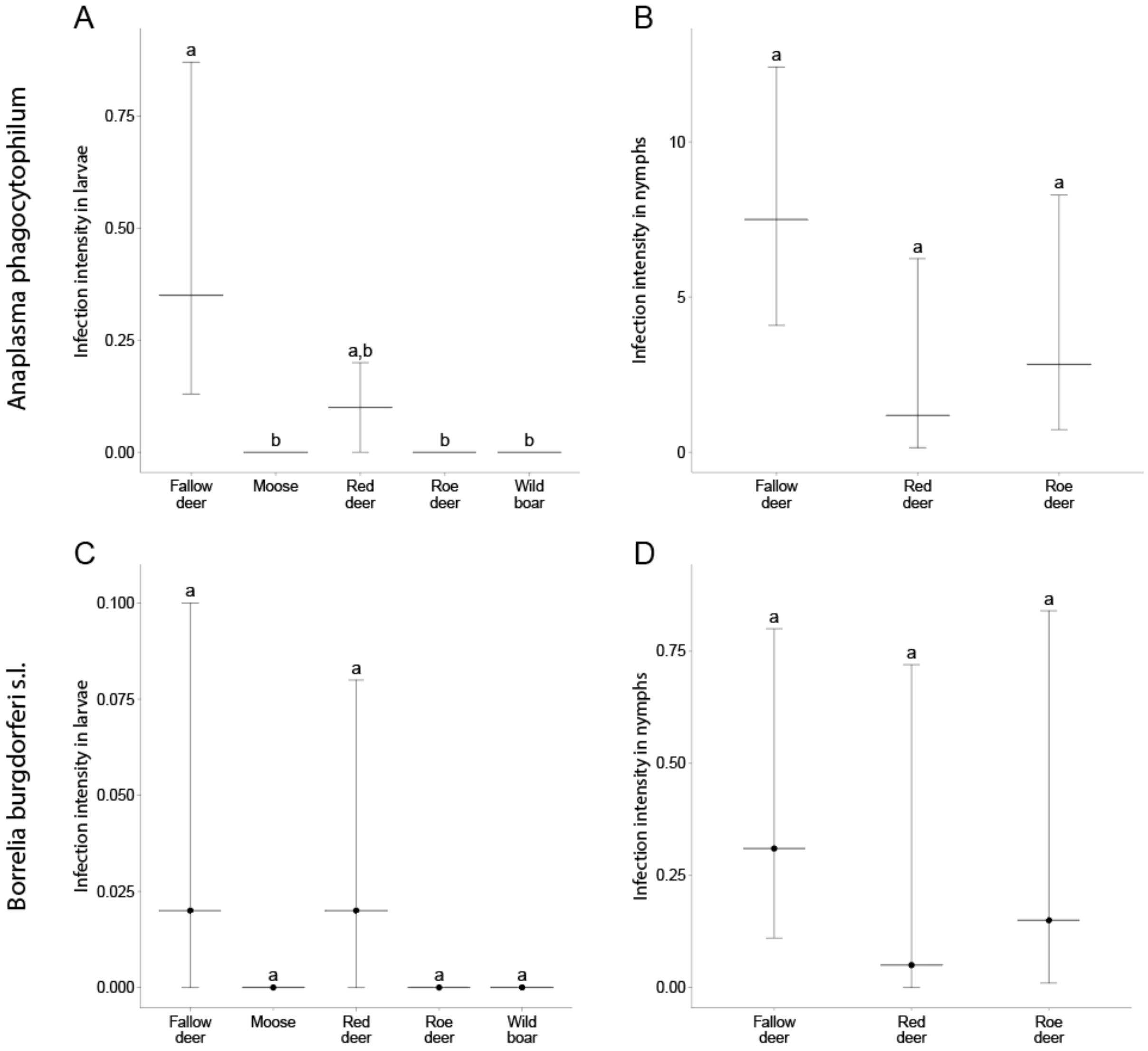

Figure S3. Infection intensity in larvae and nymphs from the studied ungulate species. Infection intensity, as calculated by formula 2, is given with 84% bootstrapped, bias-corrected, confidence intervals to show differences among ungulate species with a significance with an alpha value of 0.05. The four graphs show the *Anaplasma phagocytophilum* infection intensity in larvae (A) and nymphs (B) and the *Borrelia burgdorferi* s.l. infection intensity in larvae (C) and nymphs (D).
